# Biochemical and immunohistochemical analysis of tissue inhibitor of metalloproteinases-1 in human sound dentin

**DOI:** 10.1007/s00784-021-03819-6

**Published:** 2021-02-10

**Authors:** Pietro Gobbi, Tatjana Maravic, Allegra Comba, Claudia Mazzitelli, Edoardo Mancuso, Mirella Falconi, Lorenzo Breschi, Annalisa Mazzoni

**Affiliations:** 1 Department of Biomolecular Sciences, Carlo Bo Urbino University, Via Aurelio Saffi 2, 61029 Urbino, Italy; 2 Department of Biomedical and Neuromotor Sciences, DIBINEM, University of Bologna - Alma Mater Studiorum, Via San Vitale 59, 40125 Bologna, Italy

**Keywords:** Biochemical analysis, Immunohistochemical analysis, MMPs, TIMPs

## Abstract

**Objectives:**

Matrix metalloproteases (MMPs) are a family of enzymes that operate a proteolytic activity at the level of the extracellular matrix. MMPs are regulated by tissue inhibitors of metalloproteinases (TIMPs) that can ubiquitously bind different enzyme forms. The study aims to identify a morfo-functional association between TIMP-1 and MMP-2 and -9 in human dentin.

**Materials and methods:**

Proteins were extracted from demineralized human sound dentin powder and centrifuged to separate two aliquots with different molecular weights of proteins, higher and lower than 30 kDa. In each aliquot, the evaluation of the presence of TIMP-1/MMP-2 and TIMP-1/MMP-9 was performed using co-immunoprecipitation/immunoblotting analysis. The distribution of TIMP-1, in association with MMP-2 and -9, was investigated using a double immunohistochemical technique. Furthermore, the activity of TIMP-1 was measured by reverse zymography, where acrylamide gel was copolymerized with gelatin and recombinant MMP-2.

**Results:**

Co-immunoprecipitation/immunoblotting analysis showed the association TIMP-1/MMP-2 and TIMP-1/MMP-9 in human sound dentin. Electron microscopy evaluation revealed a diffuse presence of TIMP-1 tightly associated with MMP-2 and -9. Reverse zymography analysis confirmed that TIMP-1 present in human dentin is active and can bind different MMPs isoforms.

**Conclusions:**

The strict association of TIMP-1 with MMP-2 and -9 in situ appeared a constant finding in the human sound dentin.

**Clinical relevance:**

Considering the role of TIMP-1, MMP-2, and MMP-9 within the connective tissues, clinically applicable protocols could be developed in the future to increase or decrease the level of TIMPs in human dentin to regulate the activity of MMPs, contributing to reduce caries progression and collagen degradation.

## Introduction

Extracellular matrix metalloproteases (MMPs) are a family of Zn^2+^- and Ca^2+^-dependent proteases mainly produced by connective tissue cells. These endogenous enzymes are important components of many physiological and pathological processes due to their ability to degrade almost all proteins of the extracellular matrix (ECM) [1]. The biochemical and structural aspects of MMPs have been previously investigated in terms of activation process, catalytic mechanisms, and substrate specificity [2]. The MMPs family consists of 23 members classified in relation to substrate specificity and structure homology, further subdivided into 6 groups: collagenase, gelatinase, stromelysin, matrilisine, membrane metalloproteinases (MT-MMP), and other MMPs [2–4].

The MMPs activity is a multi-level-regulated process that includes transcription, zymogen secretion, degranulation of intracellular granules, enzymes encapsulation, and inhibitory effect of extracellular enzymes within extracellular clearance processes [3]. Tissue inhibitors of MMPs (TIMPs) are specific inhibitors of matrixines for local control of MMPs activity. The first TIMP capable of inhibiting collagenase activity was isolated from fibroblasts cells [5, 6] and named TIMP-1 with a molecular weight of approximately 28 kDa [7]. After this first identification, three new TIMPs were identified and named TIMP-2 (molecular weight of ~ 22 kDa), TIMP-3 characterized by an unglycosylated (24 kDda), and a glycosylated (27 kDa) form, and TIMP-4 with an unglycosylated form (ranging between 21 and 23 kDa) and a glycosylated (29 kDa) form [8–12].

Since the activity of MMPs is opposed by the TIMPs, the balance between these two constituents plays an important role in the maintenance of the tissue structural integrity and, as a consequence, an imbalance of this equilibrium could lead to various diseases including rheumatoid arthritis, cancer, and oral diseases such as periodontitis [13, 14]. However, although there is considerable amount of information on MMPs function and activity, only few data are available on the role and functions of TIMPs in tooth substrates.

Within the dentin-pulp complex, MMPs seem to play a pivotal role in regulating and controlling physiological processes [4, 15, 16]. However, in addition to physiological roles, it has been suggested that MMPs are involved in several pathological processes, such as periodontitis, dentin caries progression and collagen breakdown within the adhesive interface created by dentin-bonding adhesive systems [17–21]. Considerable research has been devoted to the MMP subfamily of gelatinases (i.e., MMP-2 and MMP-9). Both active and latent forms of MMP-2 have been identified in extracts from mineralized and demineralized human dentin, as well as other gelatinolytic species including several forms of MMP-9 [22–25]. TIMP-1, -2, and -3 were reported to be expressed in odontoblasts and in pulp tissue [16] and TIMP-1 has been also found to be present in pre-dentin and dentin [17, 26–28], with only one study reporting the colocalization of TIMP-1 with MMP-9 and TIMP-2 with MMP-2 [28]. However, there are no reports available on the colocalization of TIMP-1 with MMP-2.

To better clarify the role and relationship between TIMP-1 and MMP-2 and -9 in human sound dentin, this study aimed to investigate the three-dimensional and functional association between the dentin TIMP-1 and MMP-2 and MMP-9. The null hypothesis is that there is no association between TIMP-1 and MMP-2 and/or MMP-9 in human sound dentin.

## Materials and methods

All reagents were purchased from Sigma-Aldrich (Saint Louis, MO, USA), unless specified otherwise.

### Specimen preparation

Twenty-five freshly extracted sound human third molars were obtained from anonymous individuals following their signed consent under a protocol approved by the Ethical Committee of the University of Bologna, Italy (ASL_BO protocol N° 0013852 approved on 02/01/2019). Tooth crowns were cut using a low-speed diamond saw (Micromet, Remet, Bologna, Italy) under water irrigation and enamel, cement, and pulp tissue were carefully removed. Dentin fragments were crio-fractured after immersion in liquid nitrogen, pulverized using a steel hammer (Reimill, Reggio Emilia, Italy), and dentin powder was stored at −20 °C until use.

### Co-immunoprecipitation and immunoblotting

Aliquots (500 mg) of dentin powder were randomly assigned to one of the following treatments: group A: co-immunoprecipitation/immunoblotting of TIMP-1/MMP-2 and group B: co-immunoprecipitation/immunoblotting of TIMP-1/MMP-9. Each aliquot was partially demineralized in 1% H_3_PO_4_ for 10 min at 4 °C, buffered with NaOH 4N, centrifuged, and briefly washed with distilled water. Proteins of each aliquot were then extracted in extraction buffer [50 mM TRIS-HCl, 5 mM CaCl_2_, 100 mM NaCl, 0.1% Triton X-100, 0.1% NONIDET, 0.1 mM ZnCl_2_, 0.02% NaN_3_, and EDTA-free protease inhibitor cocktail (Roche Diagnostics, GmbH, Germany)] at 4 °C overnight.

**Fig. 1:**
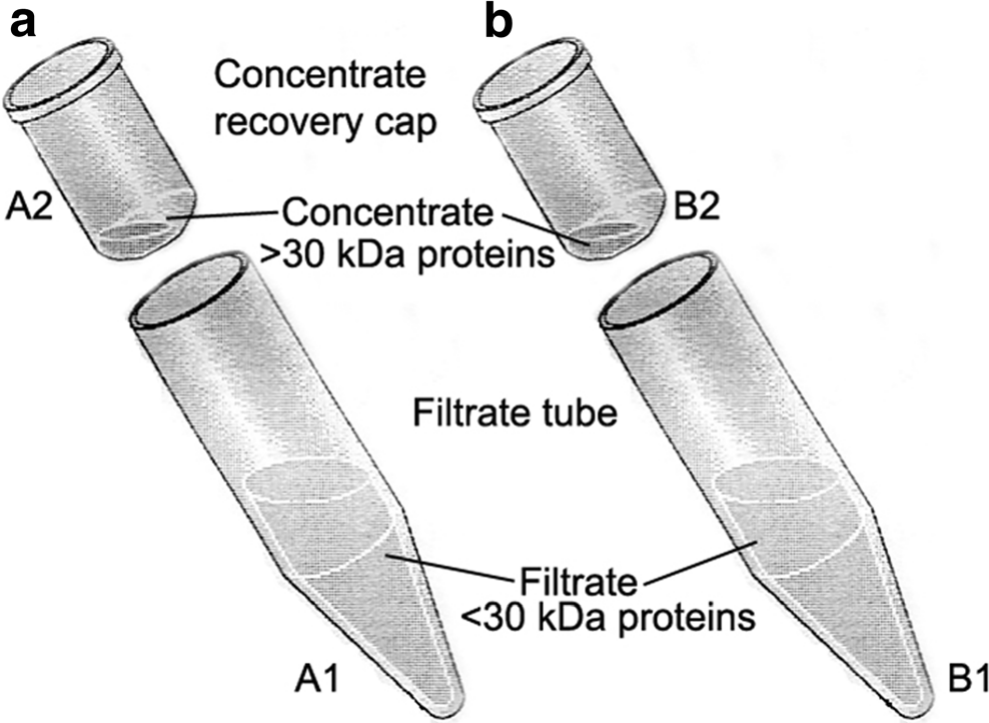
Schematic representation of proteins separation according to molecular weight. Vivaspin columns used in this study have a cut-off of 30 kDa. Aliquots A1 and B1 are concentrated proteins with a molecular weight ranging up to 30 kDa. Aliquots A2 and B2 are composed of concentrated proteins with a molecular weight ranging from 30 kDa to proteins with higher molecular weight

Extracted proteins were centrifuged with Vivaspin (cut-off 30 kDa; Vivascience Ltd, Stonehouse, UK), to obtain 2 pools of proteins: the filtered proteins with molecular weight lower than 30 kDa (groups A1 and B1) and the concentrated proteins with a molecular weight higher than 30 kDa (groups A2 and B2) (Fig. 1).

Filtered proteins (groups A1 and B1) were analyzed by western blotting to detect free forms of TIMP-1. Filtered proteins (30 μl) of both treatment groups were loaded on 12% acrylamide gel, and after electrophoretic running, proteins were transferred to nitrocellulose membrane for western blotting analysis. Membranes were stained with Ponceau red, blocked for 1.5 h with 5% bovine serum albumin (BSA) solubilized in TBS-0.1% Tween-20 (pH 7.5) and incubated overnight with primary antibody (mouse anti-TIMP-1, LOT: MAB3300, Millipore, MA, USA) diluted 1:1000 in TBS-T at 4 °C. Membranes were then washed, incubated with secondary antibody (anti-mouse IgG HRP conjugated; LOT: A9044) diluted 1:50000 in TBS-T (pH 7.5), and then incubated with ECL detection reagents (LOT: RPN2132 and RPN2133 respectively; GE Healthcare). Images were acquired with Kodak image station 2000R.

Concentrated proteins (groups A2 and B2) were used to co-immunoprecipitate TIMP-1/MMP-2 and TIMP-1/MMP-9 complexes. A primary antibody anti-MMP-2 (sc-13595, Santa Cruz Biotechnology Inc, Texas, USA) or anti-MMP-9 (IM61, Calbiochem, Darmstadt, Germany) was added to concentrated proteins and incubated overnight at 4 °C. Then, a secondary antibody (Protein A/G Plus-Agarose, sc-2003, Santa Cruz Biotechnology Inc.) was added and incubated for 4 h at 4 °C. Then, specimens were centrifuged at 14.000 rpm for 20 min, the supernatant was discarded, and proteins rinsed in PBS. Pellet was resuspended in × 2 Laemmli sample buffer, boiled for 5 min at 95 °C, and centrifuged again at 14.000 rpm for 10 min at room temperature. The supernatant was recovered and analyzed by 12% SDS-Page/immunoblotting for the detection of TIMP-1 as previously described [22].

### Immunohistochemical identification of TIMP-1/MMP-2 and TIMP-1/MMP-9 complexes

Mid-coronal dentin discs from 8 additional sound human teeth were created with a low-speed diamond saw under water irrigation (Micromet); immersed in liquid nitrogen; and crio-fractured with a steel hammer (Reimill) to produce small, smear layer-free dentin fragments [23, 24]. The selected fragments were partially demineralized with 15% citric acid (pH 1.5) at room temperature for 60 s, extensively rinsed with distilled water and processed for a pre-embedding double immunolabeling technique [23, 24, 29]. Dentin fragments were processed for TIMP-1/MMP-2 and TIMP-1/MMP-9 visualization: primary antibodies mouse IgG anti-MMP-2 (Calbiochem) and a rabbit IgG anti-TIMP-1 (Abcam, Cambridge, UK) were used to co-immunolabel TIMP-1/MMP-2, while primary antibodies mouse IgG anti-MMP-9 (Abcam) and the rabbit IgG anti-TIMP-1 (Abcam) were used to co-immunolabel TIMP-1/MMP-9.

In brief, specimens were immersed in a 0.05 M Tris–HCl buffer solution (TBS) (pH 7.6), pre-incubated in normal goat serum (British BioCell International, Cardiff, UK; dilution 1:20 in 0.05 M TBS at pH: 7.6) at room temperature, and incubated overnight with the primary antibodies (dilution 1:100 in 0.05 M TBS at pH: 7.6) at 4 °C. After rinsing in 0.05 M TBS (pH 7.6) and 0.02 M TBS (pH 8.2), gold labeling was performed with secondary antibodies goat anti-mouse and goat anti-rabbit IgG conjugated with 5- and 15-nm-diameter colloidal gold nanoparticles respectively (British BioCell International, Cardiff, UK; dilution 1:20) in 0.02 M TBS (pH 8.2) at room temperature. After immunostaining, specimens were rinsed in 0.02 M TBS (pH 8.2), fixed in 2.5% glutaraldehyde in 0.15 M cacodylate buffer (pH 7.2) for 4 h, rinsed in 0.15 M cacodylate buffer, dehydrated in ascending ethanol series (50%, 70%, 90%, 95%, 100%), and allowed to dry using hexamethyldisilasane [30]. The specimens were then mounted on stubs and carbon-coated (Balzers Med 010, Bal-Tec AG, Liechtenstein) prior to observation with a high-resolution field emission in-lens scanning electron microscope (FEI-SEM, JSM 890, JEOL Ltd., Tokyo, Japan) at 10 kV. Images were obtained using a direct analogical superimposition (mix) between the signals of the composite-backscattered electrons (BSE) and secondary electron (SE) detectors in order to achieve simultaneously a three-dimensional high-resolution image of the specimens (SE) and a clear detection of gold nanoparticles of different sizes (BSE). Controls were processed in accordance with Mazzoni et al*.* [23]: incubated overnight in 0.05 M TBS (pH 7.6) without the primary antibodies, then with the secondary antibody (data not shown).

### Reverse zymography analysis

TIMP-1 was analyzed by reverse zymography. Two aliquots of dentin powder were partially demineralized with 1% H_3_PO_4_ at 4 °C for 10 min, then proteins were extracted for zymographic assays as described by Breschi et al*.* [31]. Briefly, specimens were resuspended in extraction buffer for 24 h, sonicated and centrifuged at 14.000 rpm. Supernatants were collected and protein content was precipitated with 25% trichloroacetic acid, rinsed twice with 1 ml acetone and 0.5 ml diethyl ether, and resolubilized in loading buffer (Trizma and sodium dodecyl sulfate (SDS) in water; pH: 8.8).

Total protein concentrations of the dentin extracts were determined using Bradford assay and proteins extracted were electrophorized under nonreducing conditions on 12% SDS-polyacrylamide gels copolymerized with 2 g/L gelatin and 160 ng/ml of active recombinant MMP-2 (Calbiochem). After electrophoresis, the gels were washed twice with 2.5% Triton-X for 30 min at room temperature to remove SDS. The SDS-polyacrylamide gels were then incubated at 37 °C for 24 h in zymography buffer, stained with 0.2% Coomassie Brilliant Blue R-250, distained and scanned.

## Results

### Co-immunoprecipitation and immunoblotting

**Fig. 2:**
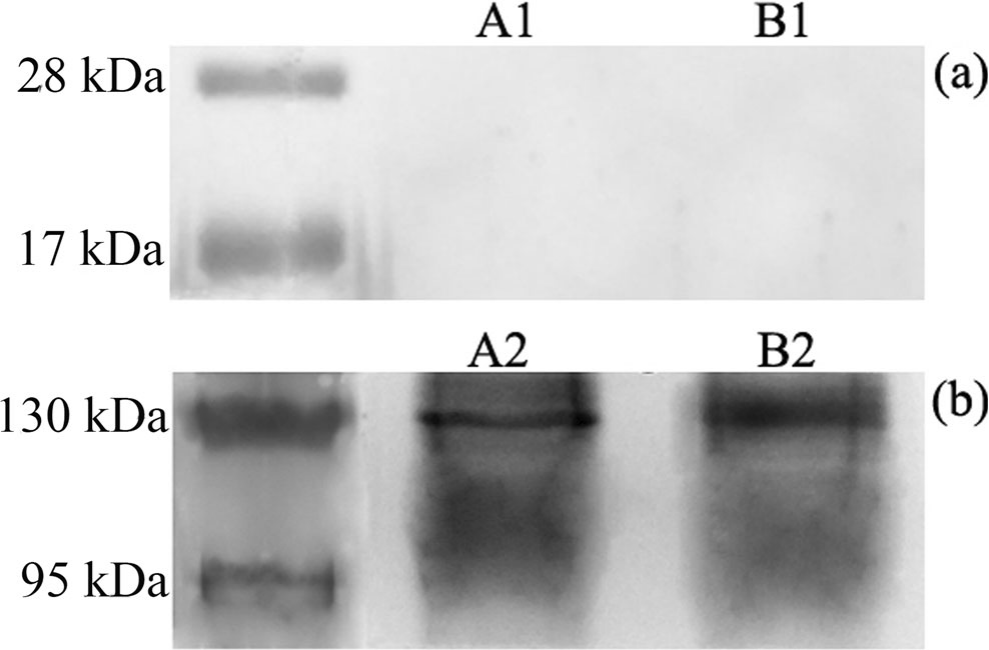
Immunoblotting analysis of TIMP-1 after co-immunoprecipitation with MMP-2 and MMP-9. **a** Filtered proteins < 30 kDa, showing the absence of TIMP-1 signal in lane A1 and B1 meaning that this regulatory protein is not present in free form within the dentin matrix. **b** Concentrated proteins with molecular weight > 30 kDA, in which TIMP-1 is detectable in association with MMP-2 (A2) and with minor intensity with MMP-9 (B2)

Western Blotting analysis of Vivaspin filtered proteins (groups A1 and B1—protein content < 30 kDa) showed absence of MMPs-free 28 kDa TIMP-1 (Fig. 2a). Conversely, western blotting analysis of concentrated proteins (groups A2 and B2—protein content > 30 kDa), processed for co-immunoprecipitation of TIMP-1/MMP-2and TIMP-1/MMP-9 to detect TIMP-1/MMPs–linked form, revealed a constant presence of the TIMP-1. The labeling intensity indicated a minor presence of TIMP-1 associated with MMP-9 (Fig. 2b).

### Immunohistochemical identification of TIMP1-/MMP-2 and TIMP-1/MMP-9

**Fig. 3:**
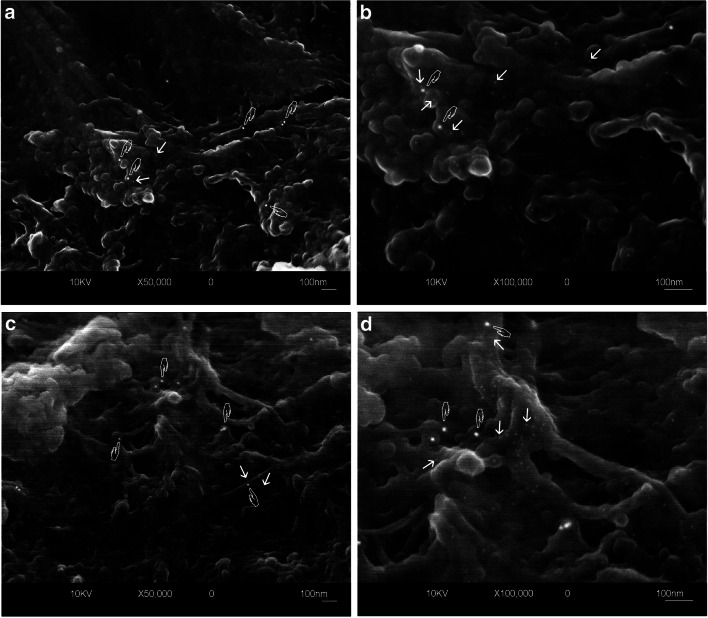

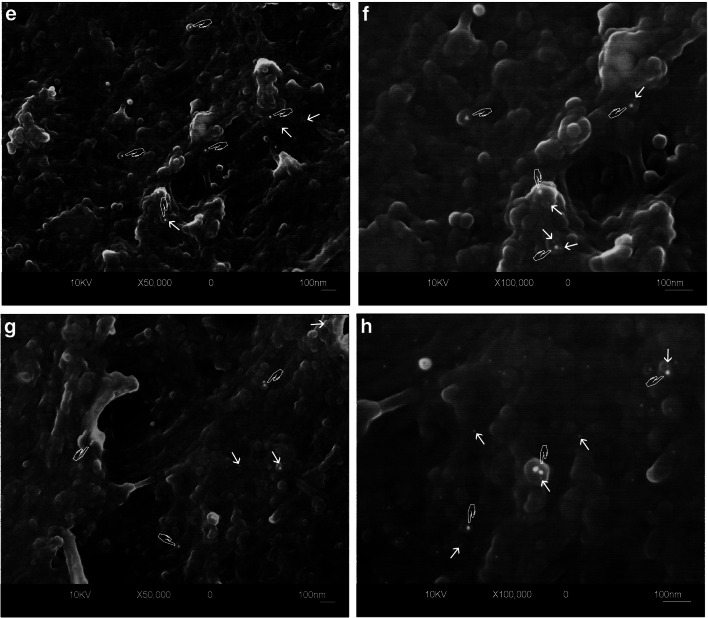
Field emission in-lens SEM (FEI-SEM) micrographs (secondary and compositional backscattered electrons image mix) of unfixed, partially demineralized dentin after immunolabeling procedure for TIMP-1/MMP-2 (**a** A, B, C, D) and TIMP-1/MMP-9 (**b** E, F,G, H). Gold particles of 5 nm (arrows), labeling for MMPs (B and D for MMP-2, F and H for MMP-9), appear widely diffused along globular structures connected to collagen fibrils at high magnification both in tubular (D, H) and intertubular (B, F) dentin. The tight spatial relationship between those small nanoparticles and 15-nm gold markers (TIMP-1 labeling) can be clearly shown (hands). At lower magnification (A = intertubular; C = tubular dentin labeled for TIMP-1 and MMP-2; E and G same areas labeled for TIMP-1 and MMP-9), although the 5-nm nanoparticles are less evident, it is still possible to appreciate a similar distribution pattern for both MMP-2 and -9. (Original magnifications × 50.000: A, C, E, G; × 100.000: B, D, F, H)

The FEI-SEM analysis showed positive immunolabeling patterns both for TIMP-1/MMP-2and TIMP-1/MMP-9 complexes revealed by the presence of 5-nm gold nanoparticles (MMP-2 or MMP-9) and of 15-nm gold particles (TIMP-1) as electron-reflective bright spherical spots on sound partially demineralized dentin surfaces (Fig. 3a and b). Labeling was distributed along the collagen fibrils, with globular structures strictly connected to the same fibrils both in the intertubular (Fig. 3aA, aB, bE, and bF) and intratubular dentin (Fig. 3aC, aD, bG, and bH). The qualitative comparison of the MMP-2 (Fig. 3aA–D) labeling patterns with respect to MMP-9 (Fig. 3bE–H) showed no substantial differences and the 15-nm-sized particles of the TIMP-1 immunolabeling appeared, at high magnification (Fig. 3aB, aD, bF, and aH), equally widely distributed. At the same high magnification pictures, a tight spatial proximity between the 15- and the 5-nm gold particles was also evident, this indicating a colocalization of TIMP-1/MMP-2 and TIMP-1/MMP-9 (Fig. 3aB, aD, bF, and bH).

Negative control specimens incubated only with the secondary antibodies revealed no immunohistochemical staining, confirming that no cross-reactions occurred between the secondary antibodies and the dentin organic matrix or with the inorganic phase (data not shown).

### Reverse zymography analysis

**Fig. 4:**
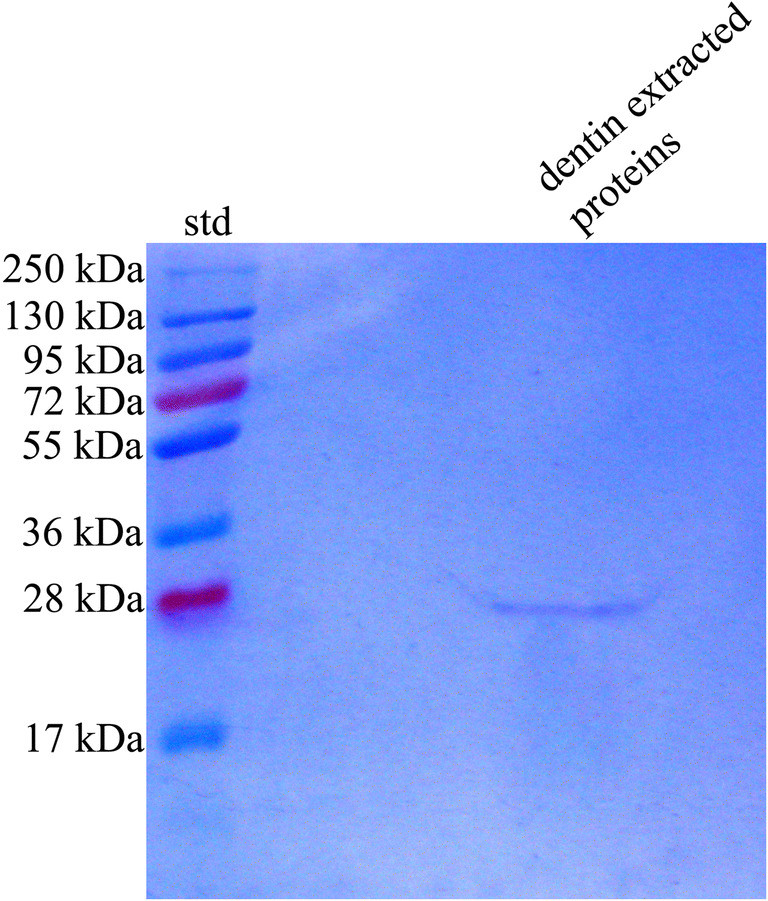
Reverse zymography analysis of partially demineralized dentin extract copolymerized with active human recombinant MMP-2. A dark band corresponding to a molecular weight of 28 kDa is easily detectable

Results of reverse zymography are shown in Fig. 4. Proteins are visible as a dark band on a light background and the extracts from partially demineralized dentin confirmed the presence of the ~ 28 kDa TIMP-1 active form associated with recombinant MMP-2, preventing its gelatinolytic activity. The molecular weight here reported is the same detected in the western blotting analyses (Figs. 2b and 4).

## Discussion

The present study aimed to perform the biochemical and immunohistochemical characterization of TIMP-1 in association with two of the major MMPs present in human dentin, MMP-2 and MMP-9. The null hypothesis has to be rejected because no free form of TIMP-1 was detectable within the sound human dentin, while TIMP-1 was found only strictly associated with MMP-2 and MMP-9. This preliminary evidence of dentin TIMP-1 led us to hypothesize that the three-dimensional association between TIMP-1 and MMP-2 and MMP-9, spatially identifiable as globular structures linked to the demineralized collagen fibrils, represents a stable endogenous component of the sound human dentin.

The results of electron microscopy analysis of co-immunolocalization of TIMP-1/MMP-2 and TIMP-1/MMP-9 provided interesting findings as the distance revealed between the colloidal gold particles for the different labeling classes of proteins is such that we actually can believe that TIMP-1 is spatially associated with MMP-2 and MMP-9 in macro molecular compounds (Fig. 3). TIMPs have been previously reported to inhibit the activity of MMPs by forming complexes with them [32], and we may speculate that the spatial proximity recorded in our study can effectively allow TIMP-1 to regulate MMP-2 and MMP-9 activity (protein’s size was checked in PDB database using Rasmol software). Furthermore, the results of the immunoblotting analysis of TIMP-1 after co-immunoprecipitation with MMP-2 and MMP-9 showed the absence of TIMP-1 signal with filtered proteins (< 30 kDa, Fig. 2a), meaning that this regulatory protein is not present in free form within the dentin matrix, while with concentrated proteins with molecular weight > 30 kDA, TIMP-1 was detectable in association with MMP-2 and with minor intensity with MMP-9 (Fig. 2b). This could be due to the fact that MMP-2 is the most abundant MMP in mature human dentin [4]. Niu et al. [28] previously investigated the association and colocalization of MMP-2 with TIMP-2, and MMP-9 with TIMP-1 using immunohistochemistry and immunofluorescence on the confocal microscope, with regard to different dentin depths. The authors reported that the concentration of the proteins significantly increased proportionally to the dentin depth. The MMP-2 and TIMP-2 were colocalized mostly in the odontoblasts while MMP-9 and TIMP-1 colocalized in odontoblasts as well as deep dentin. In the present study, gold immunolabeling enabled precise spatial correlation of the MMPs and TIMP-1 along the collagen fibrils, in the intertubular and intratubular mid-coronal dentin, overcoming the drawbacks of the immunofluorescence, such as lack of standardization and low magnification. For the first time, TIMP-1 was correlated to MMP-2, and the correlation of TIMP-1 and MMP-9 has been confirmed. These two studies cannot be fully compared due to differences in the methods used and the fact that the present study did not consider different dentin depths.

In the recent years, several studies have identified different MMPs in human dentin, such as MMP-2, -3, -9 [22], MMP-7 [33], MMP-8 [34], MMP-20 [35], and cysteine proteinases [36–38]. At the same time, important findings have been made to search for molecules that can modulate the degradation of collagen fibrils in dentin [39–41] or inhibit the action of MMPs in their proteolytic activity such as quaternary ammonium methacrylates [42, 43], chlorhexidine [44–48], and other molecules with specific targeting on MMPs [49]. These molecules have shown efficacy in MMPs inhibition and preservation of the hybrid layer over time, even up to 10 years. However, little information is present on the natural balance between MMPs and TIMPs, especially in situ within the human dentin. For this reason, the present study provided additional progress to understand the relation of TIMP-1 with human dentinal MMP-2 and MMP-9 and their distribution within the dentin matrix. The advantages of using immunohistochemical techniques using highly specific primary monoclonal antibodies, as in the present study, are mainly related to the possibility to assay precise composition and distribution of specific molecules in a sort of “biochemistry on specimens/sections” allowing to relate localization to function in situ [50].

In addition, the results of the reverse zymography allowed us to verify that the co-immunoprecipitated TIMP-1 retains their protein structure and can react with recombinant MMP-2 and may similarly react with human MMPs (Fig. 4). Reverse zymography is a technique by which protease inhibitors in a sample could be electrophoretically separated in a substrate-impregnated acrylamide gel and their relative abundance could be detected [51], as revealed in the present reverse zymography assay in which a dark band with a molecular weight of 28 kDa was detected corresponding to TIMP-1 active form (Fig. 4).

The variety of MMPs present in sound dentin play an active role during tooth development [20]. However, when the dentin matrix mineralizes, MMPs are covered with apatite nanocrystals, and probably inactivated. The mineralization of the proteases linked with their respective inhibitors could contribute to dentin structural stability [20, 52]. While it is well-known that dentin endogenous MMPs are capable to activate auto-degenerative processes within the dentin tissue [20], the results here presented demonstrate that MMPs activation is a complex cascade of events including regulative and inhibiting molecules evenly diffused within the dentin organic matrix. In fact, substrate (i.e., collagen or other non-collagenous proteins), enzymes (i.e., MMPs, cysteine cathepsins, or others), and inhibitors (i.e., TIMPs) can be simultaneously found within the dentin organic matrix, allowing the possibility of a complex enzymatic control finally contributing to the tissue homeostasis or degeneration [21].

We may speculate that clinically applicable protocols could be developed in the future to increase or decrease the level of TIMPs in human dentin as to regulate the activity of MMPs, contributing to the reduction of caries progression and collagen degradation within the adhesive interfaces created by dentin bonding systems, consequently improving the clinical outcomes of adhesive procedures in restorative dentistry.

## Conclusions

Based on the results obtained in the present study a strict association of TIMP-1 with the most represented metalloproteinases in human dentin, MMP-2 and -9, revealed to be a constant finding. However, further investigations are needed to better understand the mechanism behind the enzymatic control that contributes to the tissue balance between homeostasis and degeneration.
